# Causes and timing of death in critically ill COVID-19 patients

**DOI:** 10.1186/s13054-021-03492-x

**Published:** 2021-02-23

**Authors:** Damien Contou, Radj Cally, Florence Sarfati, Paul Desaint, Megan Fraissé, Gaëtan Plantefève

**Affiliations:** Service de Réanimation Polyvalente, Centre Hospitalier Victor Dupouy, 69, rue du Lieutenant-Colonel Prud’hon, 95100 Argenteuil, France

Mortality rate of critically ill COVID-19 patients is high, especially in those requiring invasive mechanical ventilation. However, the causes and the timing of death of patients admitted to the ICU for SARS-CoV-2 pneumonia have been poorly reported [1, 2]. Whether patients mainly die from refractory respiratory failure directly due to SARS-CoV-2 pneumonia or from sepsis as reported in non-COVID-19 ARDS patients [3] is unknown. Moreover, the increased risk of pulmonary embolism extensively described among COVID-19 patients together with the SARS-CoV-2-associated myocardial injuries [4] may expose critically ill COVID-19 patients to death from a cardiac origin [5]. Additionally, the increased intensity of thromboprophylaxis commonly used to prevent thrombotic events might also promote fatal hemorrhagic events.

We therefore aimed to describe the main causes of death among critically ill COVID-19 patients admitted to our ICU, as well as to report the timing of each cause of death.

We retrospectively reviewed all deaths occurring in adult COVID-19 patients (RT-PCR positive for SARS-CoV-2) admitted to our ICU between March 6th, 2020 and January 18th, 2021 for acute respiratory failure related to SARS-CoV-2 pneumonia.

Causes of death were categorized in four subgroups: (1) refractory respiratory failure, (2) shock with multiorgan failure, (3) cardiac death including proven pulmonary embolism (proximal thrombus on CT-pulmonary angiography with *acute cor pulmonale* on echocardiography and vasopressor requirement) and unexpected cardiac arrest (neither prior oxygen desaturation nor circulatory failure) and (4) neurological death (ischemic/hemorrhagic stroke with brain herniation).

After exclusion of COVID-19 patients still hospitalized, 152 patients were analyzed. Among them, 73 (48%, 95% confidence interval 40–56%) died with a median delay of 14 [9–23] days after ICU admission. Characteristics of the patients dying in the ICU are detailed in the Table 1.

**Table 1:** Characteristics of 73 critically ill COVID-19 patients dying during ICU stay

	COVID-19 patients dying in ICU *N* = 73
Patient’s characteristics and ICU scores	
Male sex	56 (77)
Age, years	68 [62–73]
SAPS II upon ICU admission	37 [29–45]
SOFA upon ICU admission	4 [3–8]
Main comorbidities	
Arterial hypertension	52 (71)
Diabetes mellitus	35 (48)
Ischemic cardiopathy	10 (14)
Chronic respiratory disease	18 (25)
Immunocompromised status	18 (25)
Main delays	
Days between disease onset and ICU admission	8 [6–11]
> 7 days between disease onset and ICU admission	51 (70)
Biological data upon ICU admission	
D-dimers (ng/mL)	2505 [1555–5877]
Fibrinogen (g/L)	7.3 [5.6–8.5]
Treatment administered upon ICU admission	
Glucocorticoids	35 (48)
Intermediate or full-dose thromboprophylaxis	53 (73)
Antibiotic therapy for bacterial co-infection at ICU admission	20 (27)
Antiviral drugs (lopinavir-ritonavir or remdesivir)	0 (0)
Tocilizumab	0 (0)
Outcome in the ICU	
Invasive mechanical ventilation (IMV)	71 (97)
Days between ICU admission and IMV	2 [1–4]
Days between disease onset and IMV	11 [8–14]
Ventilator associated pneumonia	47 (64)
Prone positioning	62 (85)
Extra corporal membrane oxygenation	3 (4)
Tracheostomy	2 (3)
Renal replacement therapy	26 (36)
Vasopressor support	64 (88)
Thrombotic events during ICU stay	26 (36)
Hemorrhagic events during ICU stay	17 (23)
Delay between ICU admission and death, days	14 [9–23]

Continuous variables are reported as medians [quartile 1–quartile 3] and categorical variables are reported as numbers (percentages)

*ACE/ARB* Angiotensin-Converting Enzyme Inhibitors/Angiotensin Receptor Blockers, *ICU* Intensive Care Unit, *IMV* Invasive Mechanical Ventilation, *SAPS2* Simplified Acute Physiology Score, *SOFA* Sequential Organ Failure Assessment

Distribution of the main causes of death (panel a) and timing of each cause of death (panel b) are detailed in the Fig. 1. The leading cause of death was refractory respiratory failure which accounted for 45% of ICU deaths. Cardiac deaths (all occurring in intubated patients) included 4 pulmonary embolisms (intravenous thrombolysis, *n* = 3) and 9 unexpected cardiac arrests (asystole, *n* = 7; pulseless electrical activity, *n* = 2). Neurological deaths included hemorrhagic (*n* = 4) and ischemic (*n* = 1) strokes. Overall, 10 (14%) and 6 (8%) patients directly died from a thrombotic or hemorrhagic event, respectively.

**Fig. 1:**
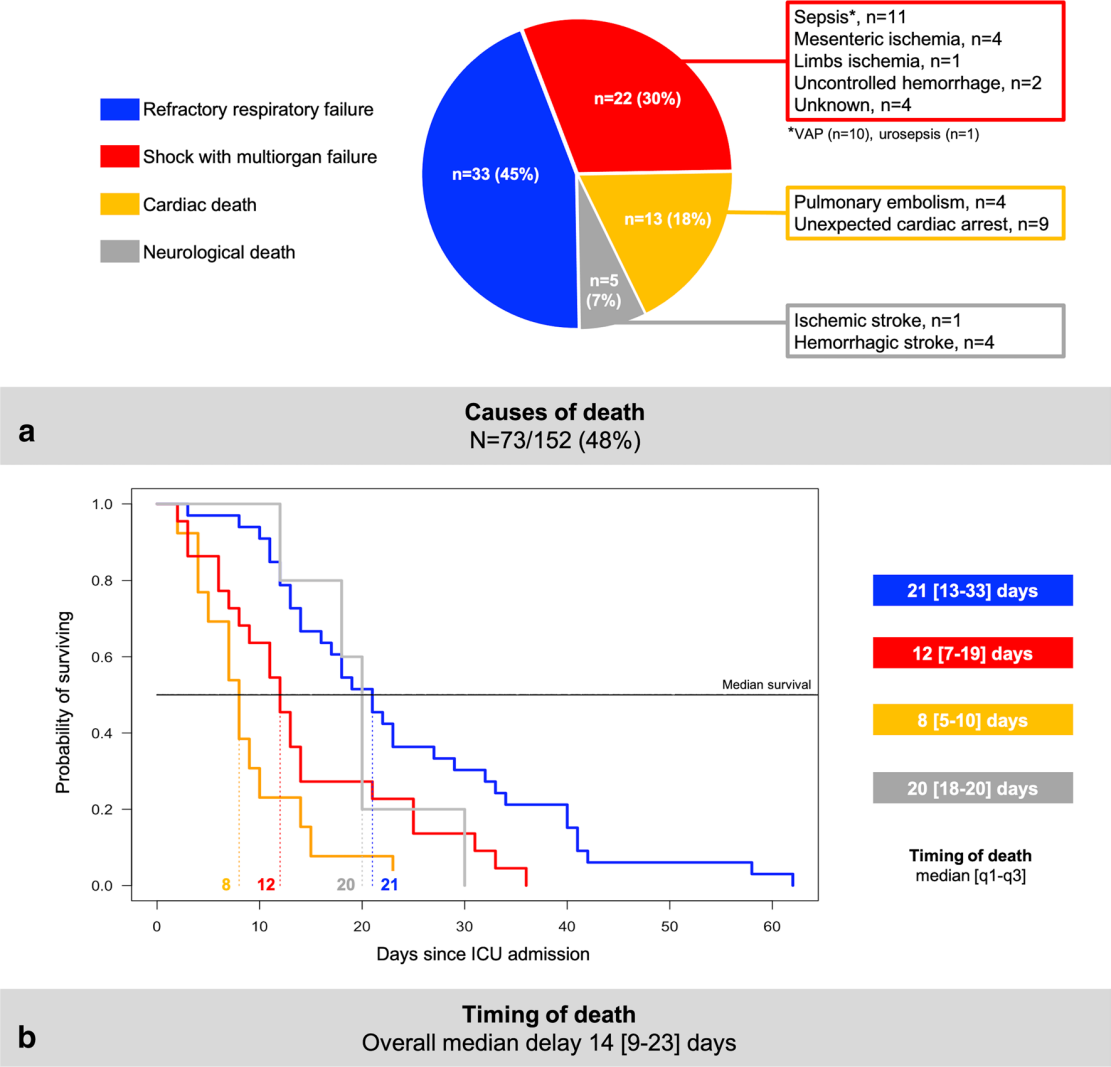
**a** Distribution of each cause of death among 73 critically ill COVID-19 patients dying during the ICU stay (*VAP* ventilator-associated pneumonia). **b** Kaplan–Meier survival estimates following ICU admission and median delay [quartile 1–quartile 3] (in days) between ICU admission and death according to each cause of death. In both panels, deaths from refractory respiratory failure, shock with multi-organ failure, cardiac and neurologic causes figure in blue, red, orange and grey, respectively

None of the patients dying from shock with multi-organ failure or from cardiac death died after a withholding (all the patients with unexpected cardiac arrest underwent cardiopulmonary resuscitation) or withdrawal procedure while all patients dying from a neurological cause died after a withdrawal procedure. Among patients dying from refractory respiratory failure, 22 (66%) and 4 died after a withholding (tracheal intubation, *n* = 2; extracorporeal membrane oxygenation, *n* = 19; renal replacement therapy, *n* = 1) or withdrawal procedure, respectively.

As opposed to non-COVID19 ARDS patients [3, 6], we herein report that refractory respiratory failure was the leading cause of death among COVID-19 ARDS patients, consistent with a previous report [2]. Deaths by refractory respiratory failure occurred late in the ICU course, potentially as a result of pulmonary fibrosis induced by SARS-CoV-2 and prolonged mechanical ventilation, making futile the use of extracorporeal membrane oxygenation support.

Noteworthy, cardiac deaths related to pulmonary embolism or unexpected cardiac arrest accounted for 18% of the deaths and occurred early in the ICU course. Similarly, a large multicenter study reported that up to 14% of the critically ill COVID-19 patients experienced cardiac arrest, mainly due to pulseless electrical activity and asystole, as a possible manifestation of fulminant myocarditis or proximal pulmonary embolism [2, 5].

Even if our study suffers from several limitations including its monocenter retrospective design, the limited number of patients and the lack of control with non-COVID-19 patients, it provides an informative picture of the main causes of death of critically ill COVID-19 patients.

## Data Availability

The dataset used and analyzed for the current study is available from the corresponding author on reasonable request.

## References

[1] Vincent J-L, Taccone FS (2020). Understanding pathways to death in patients with COVID-19. Lancet Respir Med.

[2] Ruan Q, Yang K, Wang W, Jiang L, Song J (2020). Clinical predictors of mortality due to COVID-19 based on an analysis of data of 150 patients from Wuhan, China. Intensive Care Med.

[3] Stapleton RD, Wang BM, Hudson LD, Rubenfeld GD, Caldwell ES, Steinberg KP (2005). Causes and timing of death in patients with ARDS. Chest.

[4] Guo T, Fan Y, Chen M, Wu X, Zhang L, He T (2020). Cardiovascular implications of fatal outcomes of patients with coronavirus disease 2019 (COVID-19). JAMA Cardiol..

[5] Hayek SS, Brenner SK, Azam TU, Shadid HR, Anderson E, Berlin H (2020). In-hospital cardiac arrest in critically ill patients with covid-19: multicenter cohort study. BMJ.

[6] Ketcham SW, Sedhai YR, Miller HC, Bolig TC, Ludwig A, Co I (2020). Causes and characteristics of death in patients with acute hypoxemic respiratory failure and acute respiratory distress syndrome: a retrospective cohort study. Crit Care.

